# Fabrication of UiO-66/MIL-101(Fe) binary MOF/carboxylated-GO composite for adsorptive removal of methylene blue dye from aqueous solutions†

**DOI:** 10.1039/d0ra02424d

**Published:** 2020-05-19

**Authors:** Abdelazeem S. Eltaweil, Eman M. Abd El-Monaem, Gehan M. El-Subruiti, Mona M. Abd El-Latif, Ahmed M. Omer

**Affiliations:** Chemistry Department, Faculty of Science, Alexandria University Alexandria Egypt abdelazeemeltaweil@alexu.edu.eg; Fabrication Technology Department, Advanced Technology and New Materials Research Institute (ATNMRI), City of Scientific Research and Technological Applications (SRTA-City) New Borg El-Arab City, P. O. Box: 21934 Alexandria Egypt; Polymer Materials Research Department, Advanced Technology and New Materials Research Institute (ATNMRI), City of Scientific Research and Technological Applications (SRTA-City) New Borg El-Arab City, P. O. Box: 21934 Alexandria Egypt Ahmedomer_81@yahoo.com

## Abstract

This study provides a novel composite as an efficient adsorbent of cationic methylene blue dye. UiO-66/MIL-101(Fe) binary metal organic framework (MOF) was fabricated using a solvothermal technique. Additionally, the developed binary MOF was modified with carboxylated graphene oxide (GOCOOH) using a post-synthetic technique. The as-fabricated UiO-66/MIL-101(Fe)-GOCOOH composite was analyzed by FTIR, XRD, SEM, BET, TGA, XPS and zeta potential analysis. The adsorption performance of UiO-66/MIL-101(Fe)-GOCOOH composite was examined for its aptitude to adsorb cationic MB dye using a batch technique. The obtained data revealed that, the developed UiO-66/MIL-101(Fe)-GOCOOH composite exhibited higher adsorption capacity compared to UiO-66/MIL-101(Fe) binary MOF. Adsorption isotherms and kinetic studies revealed that MB dye adsorption onto UiO-66/MIL-101(Fe)-GOCOOH composite fitted a Langmuir isotherm model (*q*_m_ = 448.71 mg g^−1^) and both pseudo 1^st^ order and pseudo 2^nd^ order kinetic models. An intra-particle diffusion model showed that the adsorption process occurs through three steps. Besides, thermodynamic data reflected that the adsorption of MB onto UiO-66/MIL-101(Fe)-GOCOOH composite is an endothermic and spontaneous process and the adsorption involves both physisorption and chemisorption interactions. The as-fabricated UiO-66/MIL-101(Fe)-GOCOOH composite exhibited good reusability and can be considered as a promising reusable adsorbent for the treatment of dye-containing industrial effluents with high efficiency.

## Abstract 2

This study provides a novel composite as an efficient adsorbent of cationic methylene blue dye.

## Graphical abstract

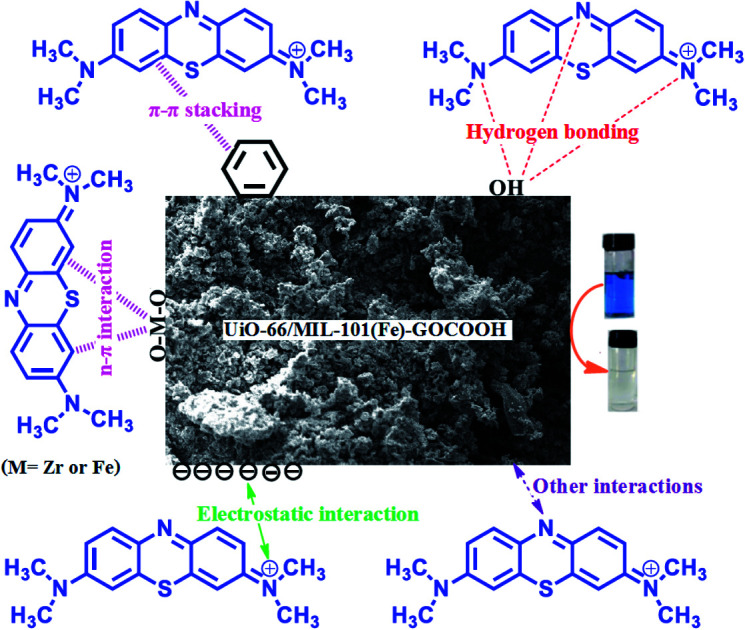

## 1. Introduction

A dye is an organic compound constructed from two main components; a chromophore that is responsible for producing the color and an auxochrome that increases the water solubility.^1,2^ Undoubtedly, dyes are a double-edged sword, since they are essential for important industries such as textiles, pharmaceuticals, plastics, polymers, refineries, and leather.^3,4^ However, the released effluents from these dye-containing industries cause significant hazards to human health and the aquatic environment. For instance, methylene blue (MB) dye is mostly utilized for coloring cotton, wood and silk, but its discharge into water bodies even in low concentration leads to various harmful impacts such as eye burning, vomiting, cyanosis, convulsions, tachycardia and methemoglobinemia.^3,5^ Accordingly, several techniques have been applied for dye removal such as advanced oxidation,^6^ membrane separation,^7^ electrolysis,^8^ catalytic reduction,^9,10^ photocatalytic degradation,^11^ flocculation.^12^ Other than the mentioned techniques, visible light-driven photocatalysis and adsorption methods are considered the most promising technologies in the environmental remediation field owing to their high efficiencies, low cost, minimal harmful by-products and their low energy consumption.^13–16^ Metal–organic frameworks (MOFs) are a blossoming category of hybrid porous materials constructed from the assembly of metal centers with organic linkers.^17^ MOFs have gained special interest because of their tunable pore size, large surface area and thermal stability.^18,19^ These unique features make MOFs an excellent candidate for industrial applications such as catalysis,^19^ drug delivery,^20^ gas storage^21^ and water treatment.^22^ One of the great advances in reticular chemistry was the MIL-101 MOF, since it exhibited high chemical stability, high surface area, remarkable thermal robustness and persistent porosity.^23^ Therefore, MIL-101 MOF has been frequently utilized for many applications including gas storage,^24^ catalysis,^25^ sensing^26^ and adsorption.^27^ Furthermore, UiO-66 is a terephthalic acid-based MOF having both tetrahedral and octahedral cavities.^28^ UiO-66 possesses high thermal and chemical stability, extremely high specific surface area, as well as, excellent adsorption properties.^29^ In the last decade, several researches have been reported on the modifications of MOFs by diverse techniques such as fabrication of bi-metallic MOFs,^30^ MOF-based composites,^31^ and binary MOFs.^32^ Although, binary MOFs have shown superior behavior in several applications such as catalysis^32^ and wastewater treatment^28^ compared with the pristine MOFs, there are few researches that have been studied the fabrication of binary MOFs and their applications. Additionally, MOF-based composites such as MIL-101(Fe)@PDopa@Fe_3_O_4_,^33^ Fe_3_O_4_/MIL-101(Fe),^34^ α-Fe_2_O_3_@UiO-66,^28^ and BiOI@UIO-66(NH_2_)@g-C_3_N_4_ (ref. 35) have been developed to overcome the limitations of MOFs.

Graphene oxide (GO), the oxidation product of graphene, contains numbers of oxygen-functional groups including hydroxyls, epoxides, carboxyls and carbonyls which significantly improve the chemical reactivity of GO compared to the raw graphene.^36^ GO has gained great concern due to its features such as large specific surface area, good mechanical characteristics, easy functionalization and its high adsorption capacity for dyes and heavy metals.^37^ Further, GO structure has been modified *via* functionalization processes which resulting in well-dispersion and high stability in aqueous solution in order to enhances its adsorption propertied and widen its applications range.^14,38^ For example, adsorption properties of MIL-101(Fe) greatly enhanced after its modification with GO.^39^

Herein, MIL-101(Fe)/UiO-66 binary MOF was synthesized *via* one-pot synthesis and then further modified by GOCOOH *via* a post-synthetic step in order to generate extra negatively charged groups and to provide a multi-functional group template with variety of available adsorption sites. The capability of the developed UiO-66/MIL-101(Fe)-GOCOOH composite for MB dye removal was estimated and discussed. Besides, isotherms, kinetics and thermodynamics of the process and reusability of UiO-66/MIL-101(Fe)-GOCOOH composite were evaluated.

## 2. Experimental

### 2.1. Materials

Graphite powder was derived from Alfa-Aesar Co. (UK). Potassium permanganate (≥99%), sodium nitrate (≥99%), zirconium oxychloride (99.5%), sulphuric acid (97%) and hydrochloric acid (37%) were supplied by Rankem (India). Hydrogen peroxide (35%), *N*,*N*-dimethyl formamide (99%), ethanol (99%) and methanol (99.9%) were bought from Merck. Chloroacetic acid (99.5%) and sodium hydroxide (97%) were obtained from Loba Chemie Ltd (India). Ferric chloride hexahydrate (99%), 1,4-benzene dicarboxylic acid (98%) and methylene blue dye were supplied by Merck.

### 2.2. Synthesis of carboxylated graphene oxide (GOCOOH)

GO was prepared *via* Hummers' method with a slight modification.^40^ In brief, 2 g graphite and 1 g sodium nitrate were dissolved into concentrated H_2_SO_4_ (100 mL) under stirring at 5 °C. Then, 10 g potassium permanganate was added and the mixture was stirred for a further 1 h. The solution temperature was raised to 40 °C and kept for 30 min under stirring. Thereafter, 100 mL deionized water was poured into the mixture and then the temperature was raised to 90 °C for 2 h. The termination of the reaction was made by adding 280 mL deionized water and 30 mL hydrogen peroxide. Finally, the brown product was separated, washed three times with HCl (10%) then distilled water and dried in oven at 50 °C overnight. In order to prepare GOCOOH, GO was soaked in 100 mL deionized water (2 mg mL^−1^) and sonicated for 30 min, then NaOH (5 g) and Cl–CH_2_COOH (5 g) were added to the suspension and followed by sonication for further 2 h. Thereafter, the solution was neutralized using HCl (10%). The obtained black solid was collected by centrifugation then washed with deionized water and methanol. At last, the GOCOOH was dried for 12 h at 50 °C.

### 2.3. Synthesis of UiO-66/MIL-101(Fe) binary MOF

UiO-66/MIL-101(Fe) binary MOF was prepared in one-pot synthesis according to the reported solvothermal method^28^ with slight modifications. In brief, 0.079 g FeCl_3_·6H_2_O was dissolved in 7.5 mL DMF solution then mixed with H_2_BDC solution (0.1626 g, 7.5 mL DMF), then stirred at 70 °C for 4 h and nominated as solution A. Subsequently, in a similar manner solution B has been prepared by dissolving 0.097 g ZrOCl_2_ and 0.05 g H_2_BDC into DMF followed by stirring at 70 °C for 4 h. After that, solutions A and B were transferred into 150 mL Teflon sealed-autoclave and kept at 130 °C for 20 h. After reaction completion, the obtained solid particles were centrifuged and washed with DMF and methanol then dried at 90 °C for 24 h.

### 2.4. Fabrication of UiO-66/MIL-101(Fe)-GOCOOH composite

The UiO-66/MIL-101(Fe)-GOCOOH composite was prepared according to the previously published procedure.^41^ Equal mass ratios of binary UiO-66/MIL-101(Fe) MOF and GOCOOH were dispersed in a certain volume of distilled water and sonicated for 30 min in order to obtain homogeneous binary MOF-GOCOOH suspensions. Then, the obtained UiO-66/MIL-101(Fe)-GOCOOH composite was separated by centrifugation and dried for 12 h at 50 °C. Three composites were synthesized depending on UiO-66/MIL-101(Fe) and GOCOOH mass ratios; UiO-66/MIL-101(Fe)-GOCOOH (1 : 1), UiO-66/MIL-101(Fe)-GOCOOH (1.5 : 0.5) and UiO-66/MIL-101(Fe)-GOCOOH (0.5 : 1.5).

### 2.5. Characterization

Samples were analyzed by Fourier transform-infrared spectra (FTIR, Shimadzu-8400S) to explore their functional groups. Thermogravimetric analyzer (TGA, Shimadzu-50) was used to evaluate the thermal behavior of the samples. Moreover, the morphological changes were clarified by scanning electron microscope (SEM, JEOL JSM 6360 LA, Japan). X-ray Phillips diffractometer was used to investigate the crystal phase. Besides, X-ray photoelectron spectroscopy (XPS, Thermo Scientific ESCALAB 250Xi VG, USA) was utilized to ivestigate the elemental composition of UiO-66/MIL-101(Fe)-GOCOOH composite surface. The textural properties were determined using the Brunauer–Emmett–Teller method (BET-Beckman coulter, SA3100, USA). Surface charges variation of UiO-66/MIL-101(Fe) binary MOF and UiO-66/MIL-101(Fe)-GOCOOH composite were determined using Zeta-sizer (Malvern-UK).

### 2.6. Batch adsorption experiment

The adsorption behavior of the synthesized UiO-66/MIL-101(Fe)-GOCOOH composite was evaluated *via* adsorption of MB dye as a model of cationic dyes. A known dose of UiO-66/MIL-101(Fe)-GOCOOH composite (0.001–0.03 g) was added to MB dye (50 to 300 mg L^−1^). pH optimization (3–11) was adjusted using 0.01 M of NaOH and/or 0.01 M of HCl. The adsorption process was conducted at various temperatures (25–55 °C) under constant agitation rate (250 rpm). The amount of un-adsorbed MB was measured spectrophotometrically at 663 nm after separation of UiO-66/MIL-101(Fe)-GOCOOH composite by centrifugation. Equations ((1) and (2), respectively) were used to calculate adsorption capacity and removal (%).1
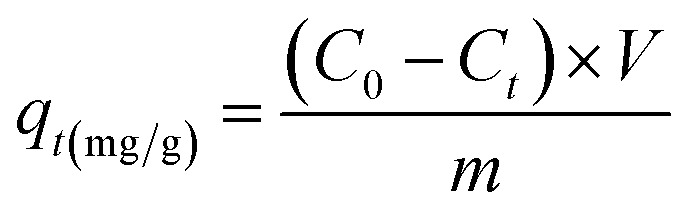
2
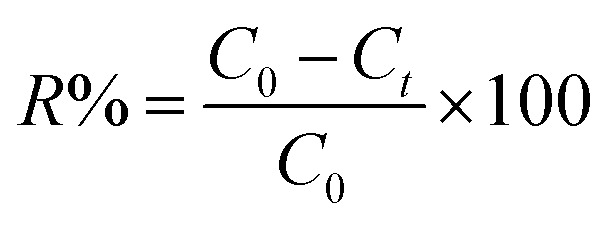
where, *C*_0_, *C*_*t*_ (mg L^−1^) symbolize MB dye concentration at zero and *t* time, respectively. *V* (L) MB volume and *m* (g) symbolizes the UiO-66/MIL-101(Fe)-GOCOOH composite mass.

**Fig. 1:**
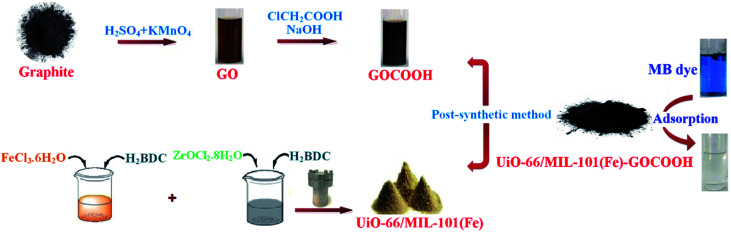
Graphical diagram for the synthesis of UiO-66/MIL-101(Fe)-GOCOOH composite and photos for adsorption process.

Fig. 1 represents a schematic diagram for the fabrication of UiO-66/MIL-101(Fe)-GOCOOH composite and laboratory images for MB before and after adsorption.

### 2.7. Reusability

To check the reusability of the synthesized UiO-66/MIL-101(Fe)-GOCOOH composite, a series of seven successive adsorption–desorption cycles was performed. After complete adsorption of MB dye, UiO-66/MIL-101(Fe)-GOCOOH composite was easily separated by centrifugation, washed with ethanol (99%) as a desorption medium, dried in air oven at 60 °C for 3 h and then tested for the next adsorption run.

## 3. Results and discussion

### 3.1. Characterization of UiO-66/MIL-101(Fe) and UiO-66/MIL-101(Fe)-GOCOOH composite

#### 3.1.1. FTIR

**Fig. 2:**
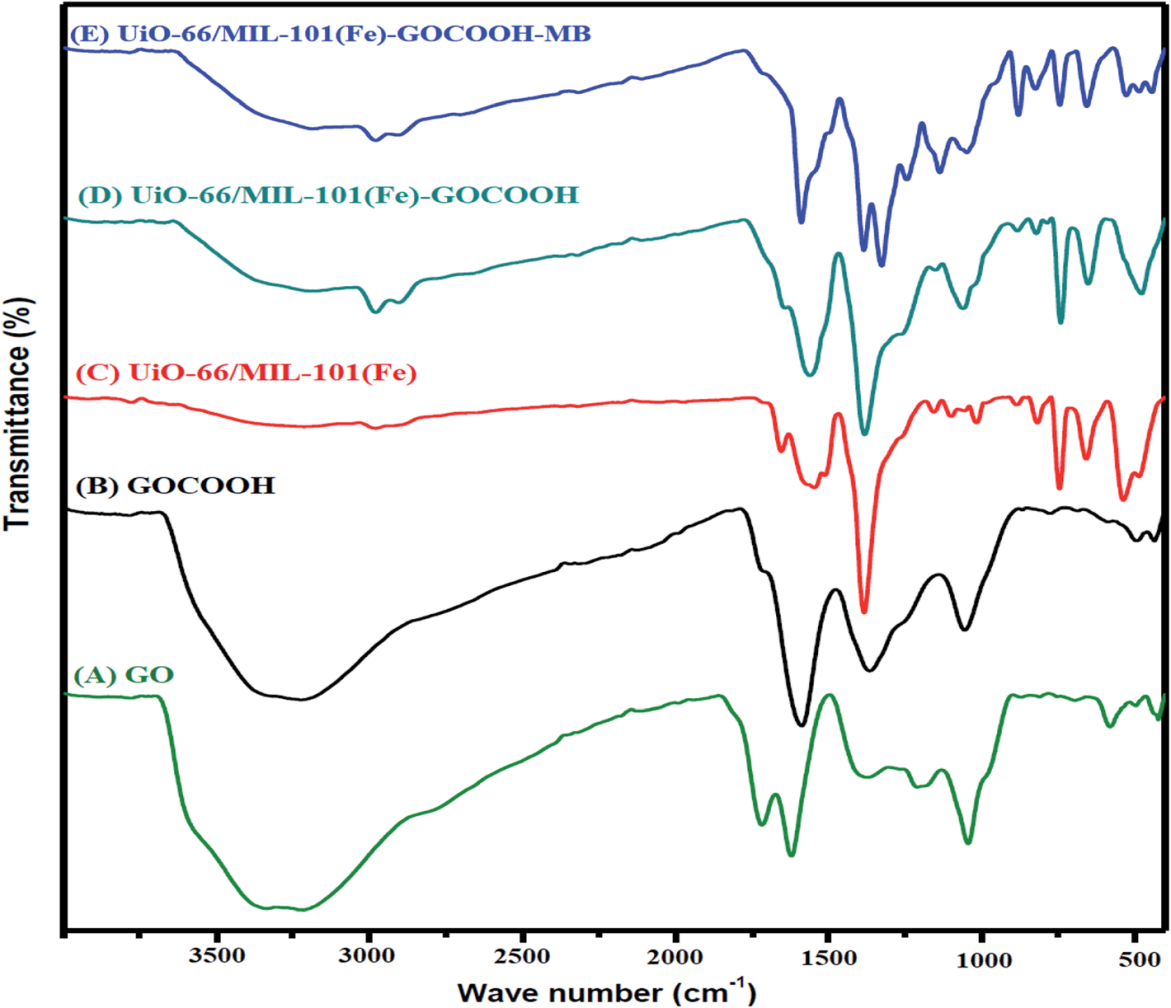
FTIR spectra of (A) GO, (B) GOCOOH, (C) UiO-66/MIL-101(Fe) binary MOF, (D) UiO-66/MIL-101(Fe)-GOCOOH and (E) UiO-66/MIL-101(Fe)-GOCOOH composite after MB dye adsorption.

FTIR spectra of GO, GOCOOH, UiO-66/MIL-101(Fe) and UiO-66/MIL-101(Fe)-GOCOOH composite are presented in Fig. 2. All spectra showed a broad band between 3000–3500 cm^−1^ which is assigned to –OH stretching. FTIR spectrum of GO (Fig. 2A) showed two peaks at 1045 and 1385 cm^−1^ which are assigned to epoxy C–O and C–OH stretching, respectively.^42^ Further, the peaks at 1612 and 1724 cm^−1^ are corresponding to the stretching vibration of C=C and C=O, respectively.^1^ Moreover, in GOCOOH spectrum (Fig. 2B) the peak at 1039 cm^−1^ is corresponding to C–O stretching and the peak at 1714 cm^−1^ is corresponding to C=O stretching, while the two peaks at 1353 and 1577 cm^−1^ are corresponding to asymmetric and symmetric vibrating bands of COOH.^43^ Besides, FTIR spectrum of UiO-66/MIL-101(Fe) binary MOF (Fig. 2C) shows a peak at 486 cm^−1^ which is ascribed to the vibration mode of Zr–O, while the peak at 549 cm^−1^ is assigned to Fe–O vibration.^44^ The peaks at 737 and 862 cm^−1^ are related to the bending vibration of aromatic C–H of benzene ring and the peak at 1120 cm^−1^ is attributed to C–C bond. Additionally, the symmetric stretching band at 1390 cm^−1^ and the two asymmetric stretching bands at 1525 and 1691 cm^−1^ are corresponding to carboxyl group.^45^ Also, the interaction between Zr and Fe ions with the de-protonated carboxyl group was confirmed by the peaks at 1525 and 1691 cm^−1^, respectively.^46,47^ Upon incorporation of GOCOOH into UiO-66/MIL-101(Fe) binary MOF, all peaks intensities decreased with the appearance of a new peak at 1060 cm^−1^ which is corresponding to C–H of GOCOOH (Fig. 2D). This confirms the successful combination between UiO-66/MIL-101(Fe) binary MOF and GOCOOH. After MB adsorption (Fig. 2E), new peaks at 1384 and 1327 cm^−1^ were noticed which are related to the aromatic rings of MB dye. Further, the C–H bond vibrations of MB dye at 874, and 1240 cm^−1^ were also observed. These new peaks and the variation in peaks intensity confirm the adsorption of MB dye onto UiO-66/MIL-101(Fe)-GOCOOH composite.

#### 3.1.2. TGA

**Fig. 3:**
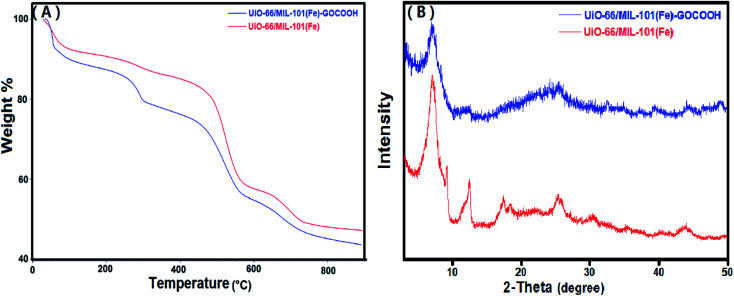
(A) TGA curves and (B) XRD patterns of UiO-66/MIL-101(Fe) binary MOF and UiO-66/MIL-101(Fe)-GOCOOH composite.

Thermal behaviors of UiO-66/MIL-101(Fe) binary MOF and UiO-66/MIL-101(Fe)-GOCOOH composite were studied using TGA. It is clear from TG curves (Fig. 3A), that both samples show three stages of weight loss. The first one between 30 and 100 °C is due to the vaporization of adsorbed water, while the second stage between 100 and 320 °C is attributed to the elimination of DMF molecules. The third weight loss starts at 450 °C is ascribed to the burning of the organic ligand which led to decomposition of both binary MOF and GOCOOH-binary MOF composite.^48^ Further, it was found that UiO-66/MIL-101(Fe)-GOCOOH composite has a total weight loss of 52.7% indicating its higher thermal stability than UiO-66/MIL-101(Fe) binary MOF which has a total weight loss of 56.5%.

#### 3.1.3. XRD

Fig. 3B shows the XRD of UiO-66/MIL-101(Fe) and UiO-66/MIL-101(Fe)-GOCOOH composite. XRD of UiO-66/MIL-101(Fe) binary MOF confirms the presence of the peaks characteristic for both UiO-66 and MIL-101(Fe) MOFs; distinguishing peaks for UiO-66 were appeared at 2*θ* = 7.17°, 17.47° and 30.37°,^49^ while the peaks at 2*θ* = 12.35°, 25.76° and 43.67° are characteristic for MIL-101(Fe).^22^ On the other hand, XRD pattern of UiO-66/MIL-101(Fe)-GOCOOH composite shows that there is no observed change in the main peaks of the binary MOF in the XRD pattern of UiO-66/MIL-101(Fe)-GOCOOH composite, however, the peaks intensity obviously decreased with no distinct peaks for GOCOOH which could be attributed to its good distribution in the sample.

#### 3.1.4. SEM

**Fig. 4:**
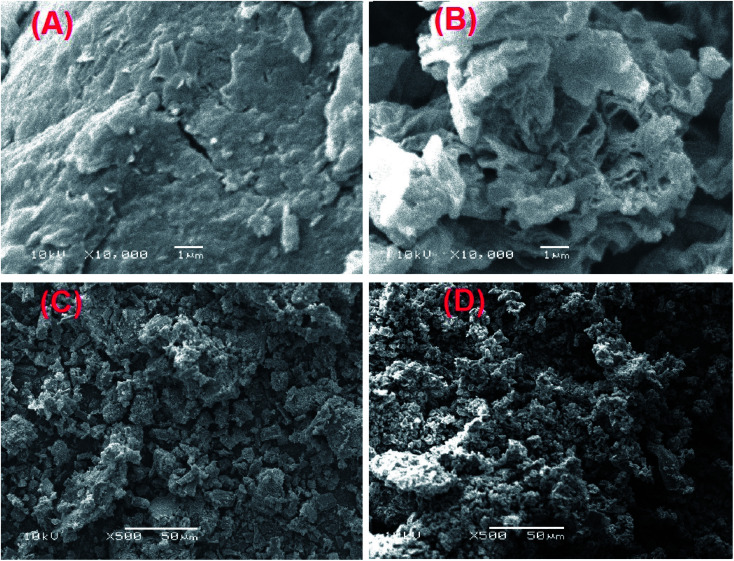
SEM images of (A) GO, (B) GOCOOH, (C) UiO-66/MIL-101(Fe) binary MOF and (D) UiO-66/MIL-101(Fe)-GOCOOH composite.

Fig. 4 represents the SEM images of GO, GOCOOH, UiO-66/MIL-101(Fe) and UiO-66/MIL-101(Fe)-GOCOOH. Fig. 4A reveals a stacking and crumpled layered structure of GO sheets, while Fig. 4B shows the crushed sheets of GOCOOH, this can be attributed to the destroying of GO sheets through the carboxylation process.^39^Fig. 4C and D clarify that UiO-66/MIL-101(Fe) and UiO-66/MIL-101(Fe)-GOCOOH particles are agglomerated and look like lumps. It can be seen that UiO-66/MIL-101(Fe) particles have a heterogeneous shape and UiO-66/MIL-101(Fe)-GOCOOH particles have preponderantly spherical shape.

#### 3.1.5. XPS

**Fig. 5:**
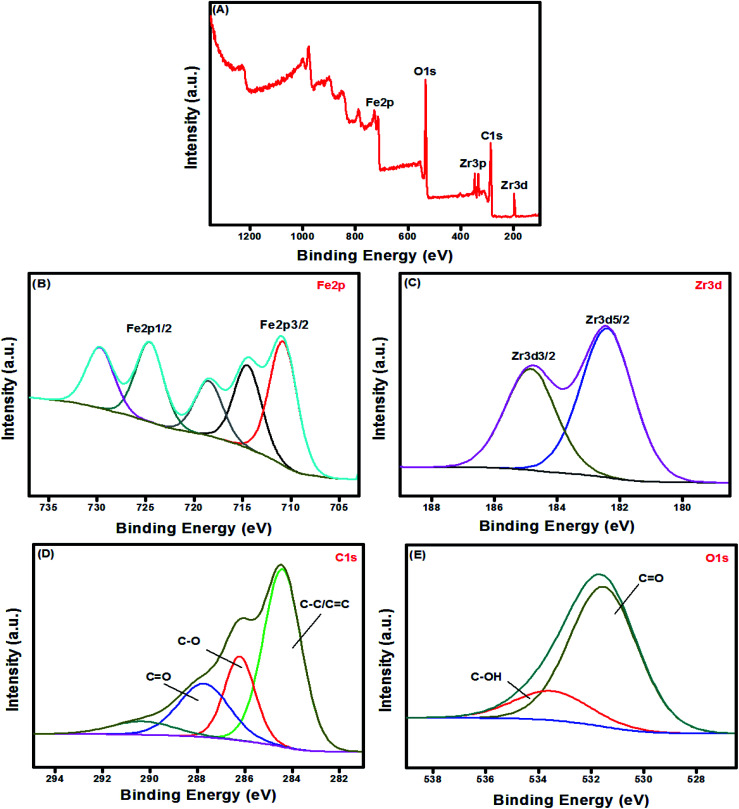
XPS spectra of (A) UiO-66/MIL-101(Fe)-GOCOOH composite; wide scan, (B) Fe 2p, (C) Zr3d, (D) C1s and (E) O1s.

XPS provides details about the surface constituents and binding energy of UiO-66/MIL-101(Fe)-GOCOOH composite. The wide XPS spectrum showed the peaks of Fe2p, Zr3d, Zr3p, C1s and O1s in UiO-66/MIL-101(Fe)-GOCOOH composite (Fig. 5A). Fe2p high resolution spectrum (Fig. 5B) shows signals at 710.48 and 724.48 eV which are related to 2p_3/2_ and 2p_1/2_ of Fe, respectively. Moreover, the two signals of Zr3d at 182.38 and 184.88 eV are ascribed to its 3d_5/2_ and 3d_3/2_ signals, respectively (Fig. 5C). The C1s spectrum of UiO-66/MIL-101(Fe)-GOCOOH composite (Fig. 5D) shows three peaks at 287.88, 286.26 and 284.38 eV which are corresponded to C=O, C–O and C–C/C=C, respectively. Whilst, the O1s spectrum (Fig. 5E) shows two signals at 531.58 and 533.76 eV ascribed to C=O and C–OH, respectively.

#### 3.1.6. BET

**Fig. 6:**
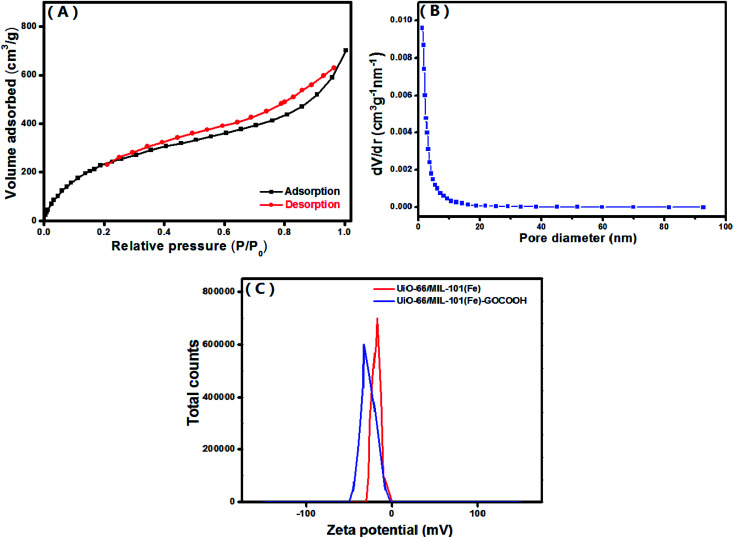
(A) N_2_ adsorption–desorption isotherm, (B) pore size distribution of UiO-66/MIL-101(Fe)-GOCOOH composite and (C) zeta potential of both UiO-66/MIL-101(Fe) binary MOF and UiO-66/MIL-101(Fe)-GOCOOH composite at pH = 7.

The textural characteristics of the UiO-66/MIL-101(Fe)-GOCOOH composite was examined by N_2_-adsorption/desorption isotherm and BJH pore size distribution (Fig. 6A and B). The BET surface area and pore volume of UiO-66/MIL-101(Fe)-GOCOOH composite are 917.24 m^2^ g^−1^ and 0.12 cm^3^ g^−1^, respectively. Moreover, the average pore sizes UiO-66/MIL-101(Fe)-GOCOOH composite is 1.22 nm.

#### 3.1.7. Zeta potential

Surface charge of the synthesized UiO-66/MIL-101(Fe) binary MOF and UiO-66/MIL-101(Fe)-GOCOOH composite (Fig. 6C) was determined using zeta potential analysis in neutral aqueous solution (pH = 7). The measured zeta potential values showed that UiO-66/MIL-101(Fe)-GOCOOH surface charge (−32.8 mV) is more negative than that of UiO-66/MIL-101(Fe) binary MOF (−17.5 mV) which can be explained by the incorporation of extra oxygen functional groups of GOCOOH.^50^ Based on these results, the adsorption capacity of the cationic MB dye onto UiO-66/MIL-101(Fe)-GOCOOH should be significantly high compared to that of UiO-66/MIL-101(Fe) binary MOF.

### 3.2. Adsorption performance

**Fig. 7:**
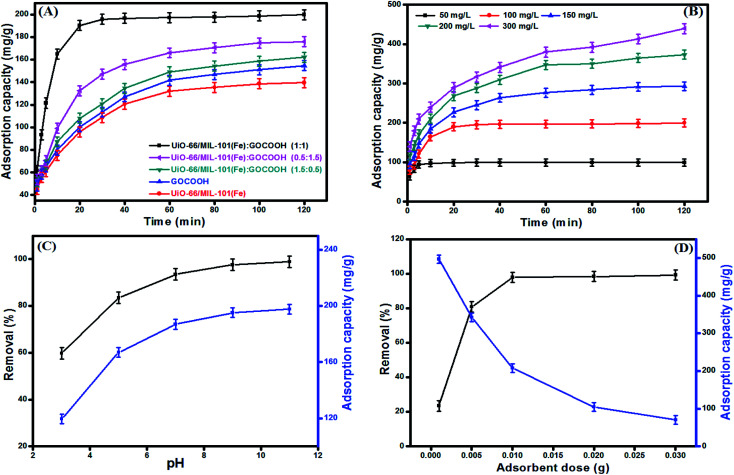
(A) Effect of GOCOOH ratio on the adsorption capacity of MB [*V*_MB_ = 20 mL, *C*_0_ = 100 mg L^−1^, *m* = 10 mg], (B) effect of initial concentration on adsorption capacity of MB onto UiO-66/MIL-101(Fe)-GOCOOH composite [*V*_MB_ = 20 mL, *m* = 10 mg, pH = 9, *T* = 25 °C], (C) effect of pH medium on removal efficiency and adsorption capacity of MB onto UiO-66/MIL-101(Fe)-GOCOOH composite [*V*_MB_ = 20 mL, *C*_0_ = 100 mg L^−1^, *m* = 10 mg, *T* = 25 °C] and (D) effect of UiO-66/MIL-101(Fe)-GOCOOH composite dosage on removal efficiency adsorption capacity of MB [*V*_MB_ = 20 mL, *C*_0_ = 100 mg L^−1^, pH = 9, *T* = 25 °C].

In order to evaluate the effect of GOCOOH incorporation on the adsorption capacity of UiO-66/MIL-101(Fe) binary MOF, a series of independent experiments were executed as following; 10 mg of each GOCOOH, UiO-66/MIL-101(Fe) binary MOF, UiO-66/MIL-101(Fe)-GOCOOH (1 : 1), UiO-66/MIL-101(Fe)-GOCOOH (1.5 : 0.5) and UiO-66/MIL-101(Fe)-GOCOOH (0.5 : 1.5) was separately added to 20 mL of MB dye (100 mg L^−1^). Fig. 7A showed that the adsorption capacity of MB onto UiO-66/MIL-101(Fe) and GOCOOH were 139.60 and 154.69 mg g^−1^, respectively. Further, the adsorption capacities of UiO-66/MIL-101(Fe)-GOCOOH (1 : 1), UiO-66/MIL-101(Fe)-GOCOOH (1.5 : 0.5) and UiO-66/MIL-101(Fe)-GOCOOH (0.5 : 1.5) were found to be 199.75, 162.13 and 175.82 mg g^−1^, respectively. The increase in the adsorption capacity upon increasing GOCOOH mass ratio from 0.33 (1.5 : 0.5) to 1 (1 : 1) could be attributed to the synergetic effect between UiO-66/MIL-101(Fe) binary MOF and GOCOOH as well as the increase in attraction forces between the positively charged MB dye molecules and the high negatively charged UiO-66/MIL-101(Fe)-GOCOOH composite resulted from the presence of extra carboxyl groups as clarified by zeta potential measurements. However, the subsequent decrease in the adsorption capacity of the composite upon increasing the GOCOOH mass ratio to 3 (0.5 : 1.5) is most likely due to the pore blocking affect, resulting from the high content of GOCOOH in the composite. A similar conclusion was reported by Y. Cao and co worker.^51^ Based on these results, the composite of equal mass ratio (UiO-66/MIL-101(Fe)-GOCOOH) was chosen for the subsequent adsorption experiments.

#### 3.2.1. Effect of initial MB dye concentration

As noticed from Fig. 7B, increasing MB initial concentration from 50 to 300 mg L^−1^ led to increasing the uptake amount (*q*_e_) of MB dye onto UiO-66/MIL-101(Fe)-GOCOOH composite from 99.51 to 439.36 mg g^−1^, this behavior is basically due to increasing the driving force that outdo the mass transfer resistance of dye molecules from bulk to the UiO-66/MIL-101(Fe)-GOCOOH surface. Otherwise, a decrease in the removal (%) from 99.5 to 68.4% was observed on increasing initial MB dye concentration which can be explained by the deficiency of active sites needed for high MB concentration.^52^

#### 3.2.2. Effect of pH

pH medium is a crucial parameter in adsorption of dyes because it controls the sign, the magnitude of the adsorbent surface charge as well as the ionization extent of dye molecules.^53^Fig. 7C demonstrated that the adsorption capacity and the removal (%) of MB dye are significantly increased with raising the pH from 3 up to 11. This behavior can be explained as follow; increasing pH of MB dye solution led to an increase in the magnitude of the negative charges on UiO-66/MIL-101(Fe)-GOCOOH composite surface, which in turn increase the electrostatic attraction between the negatively charged binary MOF-GOCOOH composite and the positively charged MB. Thus, the removal (%) and the adsorption capacity increase accordingly.

#### 3.2.3. Effect of adsorbent dosage

Fig. 7D represents the impact of the dosage on the adsorption capacity and removal (%) of MB onto UiO-66/MIL-101(Fe)-GOCOOH composite. As expected, the removal (%) of MB dye sharply increased from 23.43 to 99.2% with increasing the UiO-66/MIL-101(Fe)-GOCOOH composite dosage from 0.001 to 0.03 g; this mainly attributed to the presence of further active sites for adsorption of MB dye. Contrariwise, the adsorption capacity per unit weight of adsorbent significantly decreased from 496.66 to 70.08 mg g^−1^ with increasing UiO-66/MIL-101(Fe)-GOCOOH composite dosage from 0.001 to 0.03 g, this could be attributed to the particles aggregation of the adsorbent.^54,55^

### 3.3. Adsorption isotherm models

To investigate the nature of the interaction between the MB and the synthesized UiO-66/MIL-101(Fe)-GOCOOH composite, Langmuir and Freundlich isotherm models have been applied. The linear forms of these models are expressed by eqn (3) and (4).^56^3
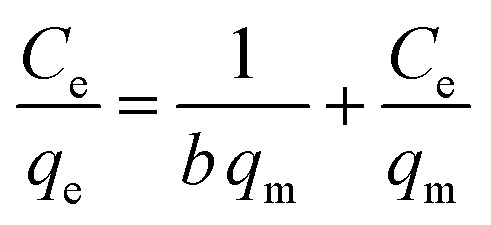
4
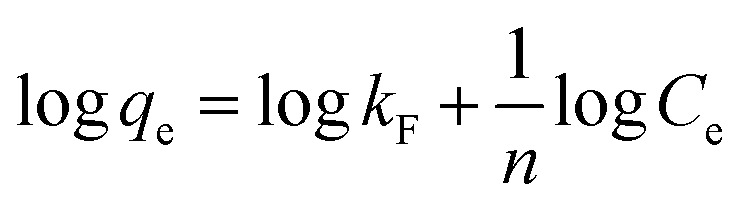
where, *q*_e_ and *C*_e_ are MB uptake amount and MB concentration at equilibrium, *b* is Langmuir constant, *q*_m_ is the theoretical maximum MB uptake, *k*_F_ and *n* are Freundlich constants.

The isotherm plots (Fig. S1†) and their derived parameters (Table 1) were clearly reflected that the experimental data best fits the Langmuir (*R*^2^ = 0.997) than Freundlich model (*R*^2^ = 0.859). Furthermore, the theoretical value of *q*_m_ (448.71 mg g^−1^) that was determined from intercept of the Langmuir plot is much close to the experimental value (439.36 mg g^−1^). Another Langmuir model-derived parameter is the dimensionless separation factor *R*_L_ (eqn (5)) that considered a credible indicator of the adsorption favorability.^57^5
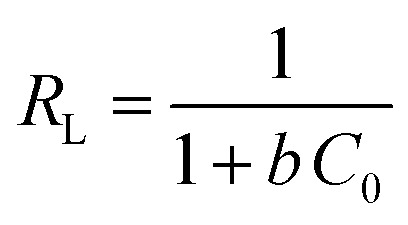
where, *C*_0_ is the initial MB dye concentration and *b* is Langmuir constant.

**Table 1:** The parameters derived from isotherm models for the adsorption of MB dye over UiO-66/MIL-101(Fe)-GOCOOH composite

Isotherms	Parameters	Value
Langmuir	*q*_m_ (mg g^−1^)	448.71 ± 5.33
	b (L mg^−1^)	0.161 ± 0.003
	*R* ^2^	0.997
Freundlich	1/*n*	0.31 ± 0.06
	*k*_F_ (mg g^−1^ (L g^−1^)^1/*n*^)	122.08 ± 2.46
	*R* ^2^	0.859

From *R*_L_ values (Table S1†), it can be deduced that the MB adsorption over UiO-66/MIL-101(Fe)-GOCOOH composite is most likely to be irreversible process where the calculated *R*_L_ values at all initial concentrations close to 0 which could be due to the strong interactions between the active sites of UiO-66/MIL-101(Fe)-GOCOOH composite and π electrons of the aromatic rings of the MB dye.^58^ Further, the *n* value obtained from Freundlich model confirmed the favorability of MB dye adsorption over UiO-66/MIL-101(Fe)-GOCOOH composite.

### 3.4. Adsorption kinetics

In order to demonstrate the rate of mechanism controlling the MB dye adsorption onto UiO-66/MIL-101(Fe)-GOCOOH composite, kinetics of adsorption was inspected by both pseudo 1^st^ order and pseudo 2^nd^ order kinetic models. The forms of these models are defined by the eqn (6) and (7), respectively. Besides, the possibility of dye particles diffusion into the UiO-66/MIL-101(Fe)-GOCOOH composite pores is elucidated by the intra-particle diffusion model presented by eqn (8).

6 ln(*q*_e_ − *q*_t_) = ln *q*_e_ − k_1_*t*

7
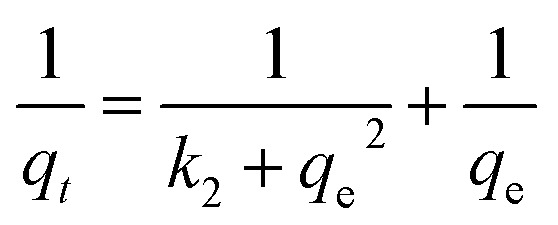

8 *q*_*t*_ = *K*_p_*t*^0.5^ + *C*

where, *q*_*t*_ and *q*_e_ are amount of MB dye uptakes at time *t* and at equilibrium, respectively, *k*_1_ and *k*_2_ are the rate constants of pseudo 1^st^ order and pseudo 2^nd^ order, respectively. *K*_p_ is the intra-particle diffusion rate constant. The values of intercept *C* provide a notion about the thickness of boundary layer.

**Fig. 8:**
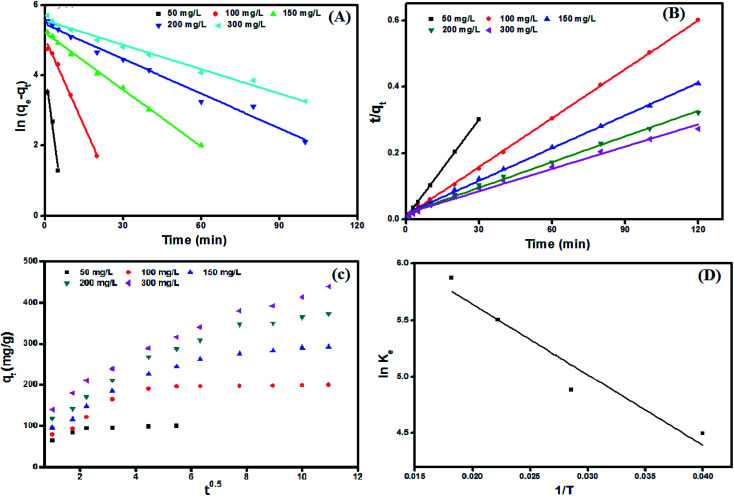
Adsorption kinetic plots; (A) pseudo first order, (B) pseudo second order, (C) intra-particle diffusion model, and (D) van't Hoff plot for adsorption of MB dye onto UiO-66/MIL-101(Fe)-GOCOOH composite.

Adsorption kinetics curves and kinetic parameters at 25 °C are represented in Fig. 8(A and B) and Table 2, respectively. The determination coefficients revealed that the adsorption kinetics is well depicted by both pseudo 1^st^ (*R*^2^ values exceeded 0.96) and pseudo 2^nd^ order (*R*^2^ values exceeded 0.99). However, the pseudo 2^nd^ order model has a higher conformity of the calculated and the experimental adsorption capacities. This means that the pseudo 2^nd^ order model provides more in-depth and rigorous reflection of the adsorption process of MB dye onto UiO-66/MIL-101(Fe)-GOCOOH composite than the pseudo 1^st^ order model does.

**Table 2:** Pseudo 1^st^ order and pseudo 2^nd^ order models parameters for the adsorption of MB dye onto UiO-66/MIL-101(Fe)-GOCOOH composite

Kinetic models	Concentration (mg L^−1^)				
	50	100	150	200	300
*q*_e,exp_ (mg g^−1^)	97.76	195.64	284.06	373.06	439.36

**Pseudo 1** ^**st**^ **order**					
*q*_e,cal_ (mg g^−1^)	64.12 ± 1.32	167.42 ± 2.42	191.13 ± 2.91	242.64 ± 3.72	290.21 ± 4.15
*k*_1_ (min^−1^)	0.557 ± 0.079	0.171 ± 0.013	0.057 ± 0.009	0.033 ± 0.003	0.024 ± 0.002
*R* ^2^	0.962	0.999	0.981	0.986	0.963

**Pseudo 2** ^**nd**^ **order**					
*q*_e,cal_ (mg g^−1^)	101.21 ± 1.11	204.08 ± 2.89	309.60 ± 3.83	389.10 ± 6.24	452.49 ± 6.41
*k*_2_ (10^−4^) (g mg^−1^ min^−1^)	200.01 ± 2.13	20.12 ± 2.02	4.04 ± 0.08	3.05 ± 0.07	2.03 ± 0.04
*R* ^2^	0.999	0.999	0.998	0.994	0.992

Intra-particle diffusion plots (Fig. 8C) showed that the MB dye adsorption onto UiO-66/MIL-101(Fe)-GOCOOH occurs throughout three steps (Fig. 2S†). Furthermore, it was observed from the intra-particle diffusion rate constants values (Table 3) that the rate of 1^st^ step > 2^nd^ step > 3^rd^ step which can be assigned to the variation in the MB dye diffusion rate during the three steps as following; in the 1^st^ step (*K*_p,1_), MB dye molecules rapidly migrate from the bulk to UiO-66/MIL-101(Fe)-GOCOOH composite surface until the outer surface of the composite become saturated. Then in the 2^nd^ step (*K*_p,2_), MB dye molecules enter the pores of UiO-66/MIL-101(Fe)-GOCOOH composite with an increase in the resistance of diffusion. Finally, in the 3^rd^ step (*K*_p,3_) MB dye molecules slowly diffuse into the pores of UiO-66/MIL-101(Fe)-GOCOOH composite up to reach equilibrium. Moreover, Fig. 8C showed that rising the initial concentration of MB dye led to an increase in the slope and intercept of all the three steps, which could be explained by the fact that the intra-particle diffusion was developed base on Fick's Law. An increase in the concentration gradient led to more rapid diffusion and faster adsorption. Also, this increase in the rate constants values for the three steps could be assigned to the increasing in the driving force of MB dye molecules that resulting from increase the initial concentration of MB dye.^59,60^ Moreover, it is clear from Fig. 8C that plots for all studied concentration do not pass through the origin (*C* ≠ 0), confirming that the intra-particle diffusion is not the only rate controlling step.^61^

**Table 3:** Intra-particle diffusion model parameters for the adsorption of MB dye onto UiO-66/MIL-101(Fe)-GOCOOH composite

*C*_0_ (mg L^−1^)	First step			Second step			Third step		
	*K*_p,1_	C_1_	*R* ^2^	*K*_p,2_	C_2_	*R* ^2^	*K*_p,3_	C_3_	*R* ^2^
50	24.28 ± 0.31	38.38 ± 0.47	0.935	1.58 ± 0.04	85.44 ± 0.96	0.989	0.32 ± 0.01	94.69 ± 1.31	0.924
100	40.69 ± 0.53	32.10 ± 0.36	0.947	3.55 ± 0.08	174.89 ± 2.21	0.888	0.81 ± 0.03	190.59 ± 2.37	0.934
150	42.56 ± 0.55	49.66 ± 0.78	0.974	19.64 ± 0.31	138.57 ± 1.84	0.994	5.33 ± 0.11	236.01 ± 2.87	0.942
200	43.82 ± 0.61	71.39 ± 1.22	0.989	21.71 ± 0.49	169.21 ± 2.06	0.990	8.47 ± 0.14	279.19 ± 3.61	0.906
300	46.55 ± 0.69	97.87 ± 1.68	0.969	27.70 ± 0.63	165.76 ± 1.97	0.999	18.78 ± 0.22	228.43 ± 2.53	0.903

### 3.5. Adsorption thermodynamics

Adsorption thermodynamics is another important section for understanding and realizing the nature and the mechanism of the MB dye adsorption process. For thermodynamic studies, the adsorption of MB dye onto the synthesized UiO-66/MIL-101(Fe)-GOCOOH composite was performed at different temperatures and the thermodynamic parameters; change in free energy (Δ*G*°), change in enthalpy (Δ*H*°) and change in entropy (Δ*S*°) were computed from the eqn (9) and (10).9
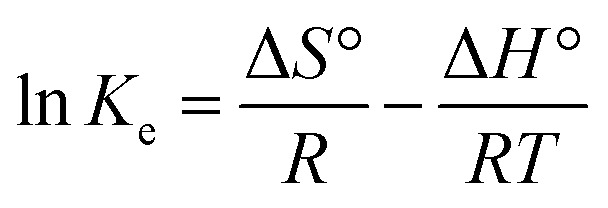

10 Δ*G*° = Δ*H*° − *T*Δ*S*°

where, *K*_e_ (*C*_Ae_/*C*_e_) is the thermodynamic equilibrium constant, *C*_Ae_ is the concentration of MB onto the UiO-66/MIL-101(Fe)-GOCOOH composite surface (mg L^−1^), *C*_e_ is the concentration of MB in solution at equilibrium (mg L^−1^), *R* is gas constant and *T* is adsorption temperature.

Δ*H*° and Δ*S*° values that have been computed from the slope and intercept of van't Hoff plot (Fig. 8D). The positive Δ*H* value reflects the endothermic nature of the adsorption of MB onto UiO-66/MIL-101(Fe)-GOCOOH composite. It has been reported that, physical adsorption is predominant when Δ*H*° value is lower than 25 kJ mol^−1^. However, chemical adsorption is predominant for Δ*H*° value ranging from 40 to 200 kJ mol^−1^.^62^ In this research, Δ*H*° is 38.53 kJ mol^−1^ which reveals that there is a chemical adsorption co-exists with the physical adsorption between UiO-66/MIL-101(Fe)-GOCOOH composite and MB dye molecules. Further, the positive Δ*S*° value reveals that the MB dye adsorption onto the synthesized UiO-66/MIL-101(Fe)-GOCOOH composite is accompanied with high randomness at solid/solution interface. Also, the negative values of Δ*G*° (Table 4) confirm that MB dye adsorption onto the synthesized binary MOF-GOCOOH composite is spontaneous process.

**Table 4:** Thermodynamic parameters for adsorption of MB dye on UiO-66/MIL-101(Fe)-GOCOOH composite

Temperature (K)	Δ*G*° (kJ mol^−1^)	Δ*H*° (kJ mol^−1^)	Δ*S*° (J mol^−1^ K^−1^)
298	−11.05	38.53	166.36
308	−12.71		
318	−14.38		
328	−16.04		

### 3.6. Mechanism of MB dye adsorption

Adsorption process of MB dye onto UiO-66/MIL-101(Fe)-GOCOOH composite is sophisticated since there are several co-existed interactions including electrostatic interactions, π–π stacking, hydrogen bonds and *n*–π conjugations, *etc.* FTIR spectra (Fig. 2E) demonstrated that the variation of peaks intensity and appearance of new peaks which strongly suggested that the removal mechanism of MB dye onto UiO-66/MIL-101(Fe)-GOCOOH composite involves electrostatic interactions between the MB dye and adsorbent functional groups which is a good agreement with pH results.^63^ Moreover, Zr–O and Fe–O centers represent good possibilities for *n*–π conjugation. This conjugation is confirmed by variation in intensity and the shift of the peaks in the range 440–530 cm^−1^. Further, kinetics data suggested the presence of physical and chemical interactions, where the adsorption of MB dye over UiO-66/MIL-101(Fe)-GOCOOH composite obeys both the pseudo 1^st^ order as well as the pseudo 2^nd^ order models. Moreover, π–π stacking mechanism is expected predicted between aromatic rings of both MB and UiO-66/MIL-101(Fe)-GOCOOH composite (originating from H_2_BDC and/or GOCOOH). In addition, the present nitrogen atoms in the structure of MB dye are expected to form hydrogen bonds with –OH groups of UiO-66/MIL-101(Fe)-GOCOOH.^64^ The possible adsorption mechanism of MB dye on the surface of UiO-66/MIL-101(Fe)-GOCOOH composite includes electrostatic interactions, π–π stacking, hydrogen bonds and *n*–π conjugations.

### 3.7. Reusability

**Fig. 9:**
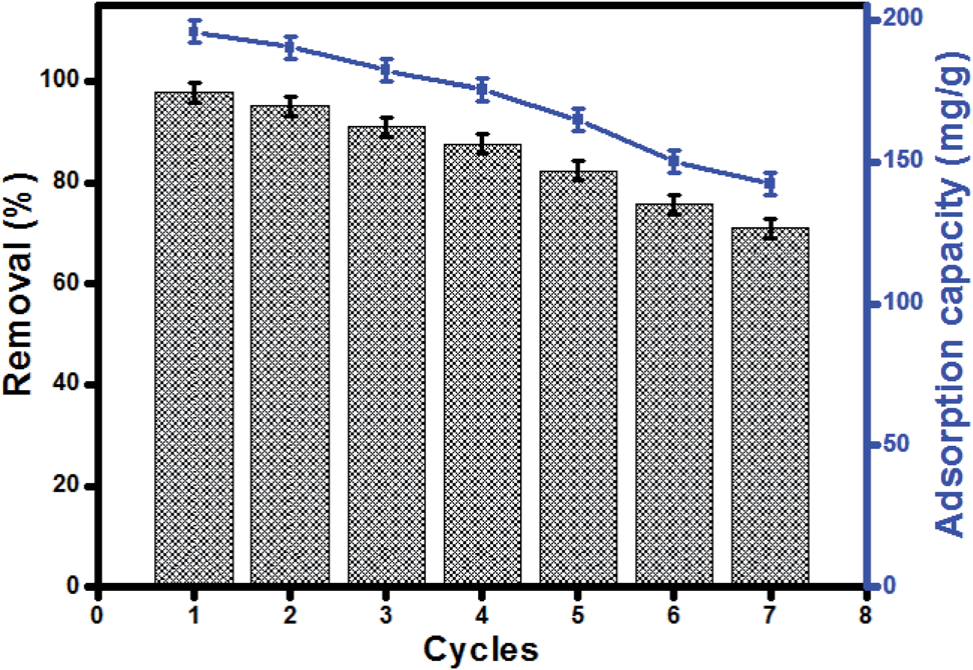
Regeneration and reusability of UiO-66/MIL-101(Fe)-GOCOOH in MB adsorption.

Reusability of UiO-66/MIL-101(Fe)-GOCOOH composite in MB adsorption was investigated for 7 cycles. The removal efficiency and adsorption capacity of MB onto UiO-66/MIL-101(Fe)-GOCOOH composite is represented in Fig. 9. A removal efficiency of 71.04% and an adsorption capacity of 142.07 mg g^−1^ were obtained after seven cycles. The reusability study indicated that UiO-66/MIL-101(Fe)-GOCOOH composite can be utilized as a re-usable adsorbent for dye removal.

### 3.8. Comparison with other adsorbents

Table 5 represented a comparison between the developed composite and some recently reported adsorbents.^65–73^ It was clear from results that UiO-66/MIL-101(Fe)-GOCOOH exhibited higher adsorption capacity for the adsorptive removal of cationic MB dye (448.7 mg g^−1^) compared with the other adsorbents. The high adsorption capacity could be a result of the successful combination between GOCOOH and UiO-66/MIL-101(Fe). Besides, the high surface area, the existence of unsaturated bonds in the fabricated composite and the generated extra negative charges on the adsorbent surface provide strong attraction forces with the positively charged MB dye molecules. In addition, the present synergetic effect of both binary UiO-66/MIL-101(Fe) MOF and GOCOOH as well as the well-dispersion of GOCOOH in the composite matrix significantly improved the adsorption process. It could be concluded that the formation of the composite allows the exceptional adsorption features of both UiO-66/MIL-101(Fe) MOF and GOCOOH to be combined which reflects positively on the adsorption process (*i.e.* boosting the adsorption capacity).

**Table 5:** Comparison of maximum adsorption capacities of MB dye onto different reported adsorbents

Adsorbent	*q*_m_ (mg g^−1^)	References
UiO-66/MIL-101(Fe)-GOCOOH composite	448.7	This study
MOF-235	252.0	65
Er-MOF	192.3	66
Ce(iii)-doped UiO-66	145.3	67
MOF1	105.0	68
Ce(iii)-doped UiO-67	398.9	69
MIL-101-SO_3_H	351.0	70
CMC/GOCOOH composite	182.3	71
rGO	144.9	72
Cu-BTC MOF/GO	152.0	73

Accordingly, UiO-66/MIL-101(Fe)-GOCOOH composite could be suggested as an efficient and reusable candidate for removing cationic dyes from their aqueous solutions.

## 4. Conclusion

A novel UiO-66/MIL-101(Fe) binary MOF-carboxylated graphene (GOCOOH) composite was fabricated and its ability for the adsorption of cationic MB dye was evaluated. Results clarified that incorporation of GOCOOH greatly increase the negative surface charge of UiO-66/MIL-101(Fe)-GOCOOH composite than the pristine UiO-66/MIL-101(Fe) binary MOF. The fabricated composite shows a superior adsorption performance for adsorbing of the cationic methylene blue dye. Moreover, the maximum adsorption capacity of MB dye onto UiO-66/MIL-101(Fe)-GOCOOH composite was found to be 448.71 mg g^−1^. Further, the recyclability test indicates a good capability of UiO-66/MIL-101(Fe)-GOCOOH to reuse for many times with no significant decrease in the adsorption capacity, confirming the application potential of our synthesized composite.

## Conflicts of interest

There are no conflicts to declare.

## Supplementary Material

RA-010-D0RA02424D-s001

## References

[cit1] Bach L. G., Van Tran T., Nguyen T. D., Van Pham T., Do S. T. (2018). Res. Chem. Intermed..

[cit2] Ye Y., Lin R.-B., Cui H., Alsalme A., Zhou W., Yildirim T., Zhang Z., Xiang S., Chen B. (2020). Dalton Trans..

[cit3] Liu C., Omer A., Ouyang X.-k. (2018). Int. J. Biol. Macromol..

[cit4] Thakur A., Kumar P., Kaur D., Devunuri N., Sinha R., Devi P. (2020). RSC Adv..

[cit5] Fil B. A., Ozmetin C., Korkmaz M. (2012). Bull. Korean Chem. Soc..

[cit6] Wu S., Lin Y., Yang C., Du C., Teng Q., Ma Y., Zhang D., Nie L., Zhong Y. (2019). Chemosphere.

[cit7] Yang G., Zhang D., Zhu G., Zhou T., Song M., Qu L., Xiong K., Li H. (2020). RSC Adv..

[cit8] Bassyouni D., Hamad H., El-Ashtoukhy E. Z., Amin N., El-Latif M. A. (2017). J. Hazard. Mater..

[cit9] El-Subruiti G., Eltaweil A., Sallam S. (2019). NANO.

[cit10] Sallam S., El-Subruiti G., Eltaweil A. (2018). Catal. Lett..

[cit11] Tan W., Luan J. (2020). RSC Adv..

[cit12] Guo K., Gao B., Tian X., Yue Q., Zhang P., Shen X., Xu X. (2019). Chemosphere.

[cit13] Tamer T. M., Hafez A. M., Roston G. D., Mohyeldin M. S., Abou-Taleb W. M., Ahmed O. (2018). Environ. Nanotechnol. Monit..

[cit14] Wang N., Wang Y.-F., Omer A. M., Ouyang X.-k. (2017). Anal. Bioanal. Chem..

[cit15] Elkady M., Shokry H., El-Sharkawy A., El-Subruiti G., Hamad H. (2019). J. Mol. Liq..

[cit16] Inthapanya X., Wu S., Han Z., Zeng G., Wu M., Yang C. (2019). Environ. Sci. Pollut. Res..

[cit17] Hu Z.-P., Gao Z.-M., Liu X., Yuan Z.-Y. (2018). Adsorpt. Sci. Technol..

[cit18] Duan M.-J., Guan Z.-y., Ma Y.-W., Wan J.-Q., Wang Y., Qu Y.-F. (2018). Chem. Pap..

[cit19] Gupta V., Tyagi S., Paul A. (2017). Integr. Ferroelectr..

[cit20] Liang R., Jing F., Shen L., Qin N., Wu L. (2015). Nano Res..

[cit21] Belmabkhout Y., Mouttaki H., Eubank J. F., Guillerm V., Eddaoudi M. (2014). RSC Adv..

[cit22] Hamedi A., Zarandi M. B., Nateghi M. R. (2019). J. Environ. Chem. Eng..

[cit23] Férey G., Mellot-Draznieks C., Serre C., Millange F., Dutour J., Surblé S., Margiolaki I. (2005). Science.

[cit24] Wang X., Liu L., Jacobson A. J. (2006). Angew. Chem., Int. Ed..

[cit25] El-Hakam S. A., Samra S. E., El-Dafrawy S. M., Ibrahim A. A., Salama R. S., Ahmed A. I. (2018). RSC Adv..

[cit26] Liu D., Lu K., Poon C., Lin W. (2013). Inorg. Chem..

[cit27] Jarrah A., Farhadi S. (2018). RSC Adv..

[cit28] Kurisingal J. F., Rachuri Y., Gu Y., Kim G.-H., Park D.-W. (2019). Appl. Catal., A.

[cit29] Taima-Mancera I., Rocío-Bautista P., Pasán J., Ayala J., Ruiz-Pérez C., Afonso A., Lago A., Pino V. (2018). Molecules.

[cit30] Singh K., Kukkar D., Singh R., Kukkar P., Bajaj N., Singh J., Rawat M., Kumar A., Kim K.-H. (2020). J. Ind. Eng. Chem..

[cit31] Han B., Zhang E., Cheng G. (2018). Appl. Sci..

[cit32] Azhar M. R., Abid H. R., Sun H., Periasamy V., Tadé M. O., Wang S. (2017). J. Colloid Interface Sci..

[cit33] Jiang Z., Li Y. (2016). J. Taiwan Inst. Chem. Engrs..

[cit34] Jiang Z. W., Dai F. Q., Huang C. Z., Li Y. F. (2016). RSC Adv..

[cit35] Liang Q., Cui S., Jin J., Liu C., Xu S., Yao C., Li Z. (2018). Appl. Surf. Sci..

[cit36] Zhang W., Zhou C., Zhou W., Lei A., Zhang Q., Wan Q., Zou B. (2011). Bull. Environ. Contam. Toxicol..

[cit37] Liu J., Chu H., Wei H., Zhu H., Wang G., Zhu J., He J. (2016). RSC Adv..

[cit38] Omer A. M., Elgarhy G. S., El-Subruiti G. M., Khalifa R. E., Eltaweil A. S. (2020). Int. J. Biol. Macromol..

[cit39] Shiva Kumar S., Ramakrishna S., Rama Devi B., Himabindu V. (2018). Int. J. Green Energy.

[cit40] Shahriary L., Athawale A. A. (2014). Int. J. Renew. Energy Environ. Eng.

[cit41] Wu Y., Luo H., Wang H. (2014). RSC Adv..

[cit42] Yang S.-T., Chen S., Chang Y., Cao A., Liu Y., Wang H. (2011). J. Colloid Interface Sci..

[cit43] Alqadami A. A., Naushad M., Abdalla M. A., Khan M. R., Alothman Z. A. (2016). J. Chem. Eng. Data.

[cit44] Wei C., Hou H., Wang E., Lu M. (2020). Materials.

[cit45] Mostafavi M. M., Movahedi F. (2018). Appl. Organomet. Chem..

[cit46] Luu C. L., Van Nguyen T. T., Nguyen T., Hoang T. C. (2015). Adv. Nat. Sci. Nanosci. Nanotechnol..

[cit47] Hu Y., Zheng S., Zhang F. (2016). Front. Chem. Sci. Eng..

[cit48] Yang Q., Zhang H.-Y., Wang L., Zhang Y., Zhao J. (2018). ACS Omega.

[cit49] Zhao W., Zhang C., Yan Z., Zhou Y., Li J., Xie Y., Bai L., Jiang L., Li F. (2017). PloS One.

[cit50] Mindivan F. (2016). Mach., Technol., Mater..

[cit51] Cao Y., Zhao Y., Lv Z., Song F., Zhong Q. (2015). J. Ind. Eng. Chem..

[cit52] Abd El-Latifa M. M., Ibrahim A. M. (2010). Desalin. Water Treat..

[cit53] Abd El-Latif M., Ibrahim A. M. (2009). Desalin. Water Treat..

[cit54] Mashkoor F., Nasar A., Asiri A. M. (2018). Sci. Rep..

[cit55] Eltaweil A., Mohamed H. A., Abd El-Monaem E. M., El-Subruiti G. (2020). Adv. Powder Technol..

[cit56] El-Sayed E., Tamer T., Omer A., Eldin M. S. M. (2016). Desalin. Water Treat..

[cit57] Wang N., Ouyang X.-K., Yang L.-Y., Omer A. M. (2017). ACS Sustainable Chem. Eng..

[cit58] Costa J. A. S., Sarmento V. H., Romão L. P., Paranhos C. M. (2019). Biomass Convers. Biorefin..

[cit59] Omer A., Khalifa R., Tamer T., Elnouby M., Hamed A., Ammar Y., Ali A., Gouda M., Eldin M. M. (2019). Int. J. Biol. Macromol..

[cit60] Omer A. M., Khalifa R. E., Hu Z., Zhang H., Liu C., Ouyang X. k. (2019). Int. J. Biol. Macromol..

[cit61] Vijayakumar G., Tamilarasan R., Dharmendirakumar M. (2012). J. Mater. Environ. Sci..

[cit62] Fan S., Wang Y., Wang Z., Tang J., Tang J., Li X. (2017). J. Environ. Chem. Eng..

[cit63] Alvarez-Torrellas S., Boutahala M., Boukhalfa N., Munoz M. (2019). Appl. Sci..

[cit64] Vargas A. M., Cazetta A. L., Kunita M. H., Silva T. L., Almeida V. C. (2011). Chem. Eng. J..

[cit65] Haque E., Jun J. W., Jhung S. H. (2011). J. Hazard. Mater..

[cit66] Mohammadnejad M., Hajiashrafi T., Rashnavadi R. (2018). J. Porous Mater..

[cit67] Yang J.-M., Ying R.-J., Han C.-X., Hu Q.-T., Xu H.-M., Li J.-H., Wang Q., Zhang W. (2018). Dalton Trans..

[cit68] Yi F. Y., Li J. P., Wu D., Sun Z. M. (2015). Chem. - Eur J..

[cit69] Yang J.-M., Yang B.-C., Zhang Y., Yang R.-N., Ji S.-S., Wang Q., Quan S., Zhang R.-Z. (2020). Microporous Mesoporous Mater..

[cit70] Luo X.-P., Fu S.-Y., Du Y.-M., Guo J.-Z., Li B. (2017). Microporous Mesoporous Mater..

[cit71] Eltaweil A. S., Elgarhy G. S., El-Subruiti G. M., Omer A. M. (2020). Int. J. Biol. Macromol..

[cit72] Minitha C., Lalitha M., Jeyachandran Y., Senthilkumar L., RT R. K. (2017). Mater. Chem. Phys..

[cit73] Jabbari V., Veleta J., Zarei-Chaleshtori M., Gardea-Torresdey J., Villagrán D. (2016). Chem. Eng. J..

