# Supplementary material for: Fabrication of UiO-66/MIL-101(Fe) binary MOF/carboxylated-GO composite for adsorptive removal of methylene blue dye from aqueous solutions
Source: RSC Adv. 2020 May 19;10(32):19008–19. doi: 10.1039/d0ra02424d (PMC9053870; doi:10.1039/d0ra02424d)
Supplement: RA-010-D0RA02424D-s001 [file RA-010-D0RA02424D-s001.pdf]

Electronic Supplementary Material (ESI) for RSC Advances.
This journal is © The Royal Society of Chemistry 2020

Fabrication of UiO-66/MIL-101(Fe) binary MOF/Carboxylated-GO composite for adsorptive removal of methylene blue dye from aqueous solutions

Abdelazeem S. Eltaweil [a,*], Eman M. Abd El-Monaem [a], Gehan M. El-Subruiti [a], Mona M. Abd El-Latif [b], Ahmed M. Omer [c,*]

Supplementary data

Table S1: R_L values for the adsorption of MB dye onto UiO-66/MIL-101(Fe)-GOCOOH composite Based on the Langmuir model.

C_o (mg/L)	R_L
50	0.11
100	0.06
150	0.04
200	0.03
300	0.02

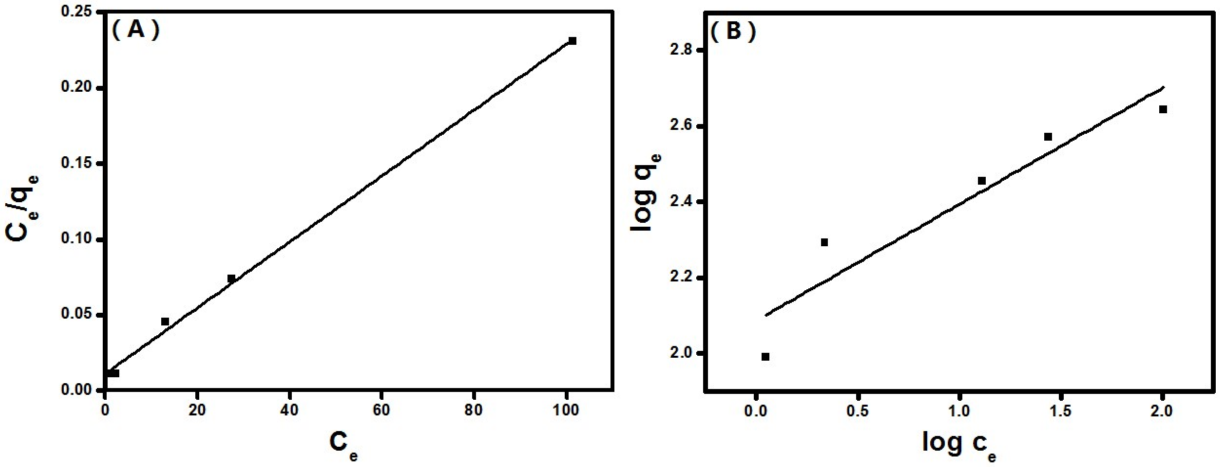

Fig. S1. Adsorption isotherm model (A) Langmuir and (B) Freundlich for the adsorption of MB dye onto UiO-66/MIL-101(Fe)-GOCOOH composite.

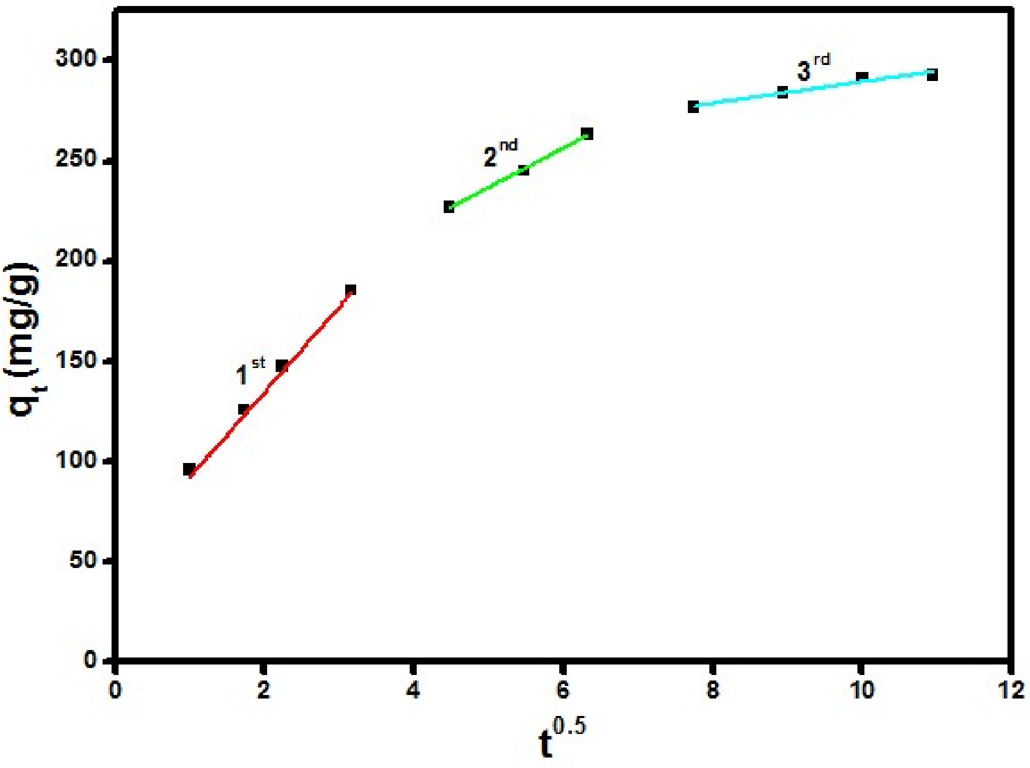

Fig. S2. intra-particle diffusion model for adsorption of MB dye onto UiO-66/MIL-101(Fe)-GOCOOH composite.
